# Whole lung lavage for pulmonary alveolar proteinosis

**DOI:** 10.4103/0970-2113.59267

**Published:** 2010

**Authors:** S. Jayaraman, A. R. Gayathri, P. Senthil Kumar, Rajeev Santosham, Rajan Santosham, R Narasimhan

**Affiliations:** Department of Respiratory Medicine, Apollo Hospitals, Chennai - 600 006, India; Santosham Chest Hospital, Chennai - 600 006, India; Department of Cardiothoracic Surgery, Apollo Hospitals, Chennai - 600 006, India

**Keywords:** Open lung biopsy, pulmonary alveolar proteinosis, whole lung lavage

## Abstract

A 26-year-old male presented with complaints of dry cough of six months and progressive breathlessness of three months duration. He was coughing out milky white sputum for two months and had lost 12 kg weight in two months. He had an evening rise in temperature of one month duration. Clinically, the patient was in respiratory distress and the respiratory system examination revealed bilateral velcro crackles. High resolution computed tomography chest showed bilateral diffuse reticulonodular opacities and “Crazy Paving” pattern suggestive of alveolar proteinosis. Broncho alveolar lavage showed eosinophilic granular material, which was periodic acid-Schiff positive. Open lung biopsy was done to confirm the diagnosis and the histopathologic examination revealed eosinophilic secretions with granular appearance suggestive of pulmonary alveolar proteinosis. Subsequently, patient underwent bilateral sequential whole lung lavage under general anesthesia. Patient showed marked clinical and radiological improvement after sequential whole lung lavage.

## INTRODUCTION

Pulmonary Alveolar Proteinosis (PAP) is a rare idiopathic lung disease characterized by the accumulation of lipoproteinaceous material within the alveoli of the lungs. This disease was first described in 1958 and till date fewer than 500 cases have been reported in medical literature. Although the pathogenesis of PAP has remained unknown, most investigators have postulated a decreased clearance of lipids and surfactant proteins from the air spaces, by the alveolar macrophages and type-2 epithelial cells. Historically, there has been no effective pharmacotherapy for PAP, and sequential whole-lung lavage under general anesthesia has become the mainstay of treatment. In fact, Whole lung lavage is the only therapy that has emerged to decrease the symptoms and improve the oxygenation in patients with PAP. The usual end point of Whole lung lavage is improvement in the clinical, physiologic and radiologic aspect of the patient.

## CASE REPORT

A 26-year-old male Engineering Student from Tamil Nadu presented with complaints of dry cough of six months and progressive breathlessness of three months duration. He had cough with milky white sputum for two months prior to presentation to the hospital. He had lost about 12 kg weight over a two-month period. He had history of evening rise of temperature for one month duration. On examination, patient was in respiratory distress and SpO_2_- at rest on air was 78% and with five liters oxygen supplementation via face mask was 91%. Respiratory system examination revealed bilateral velcro crackles. Other system examination was unremarkable. He had no other co morbid illnesses. There was no history of drug intake or exposure to toxic fumes or inorganic dust. He was a nonsmoker.

### Investigations

**Figure 1:**
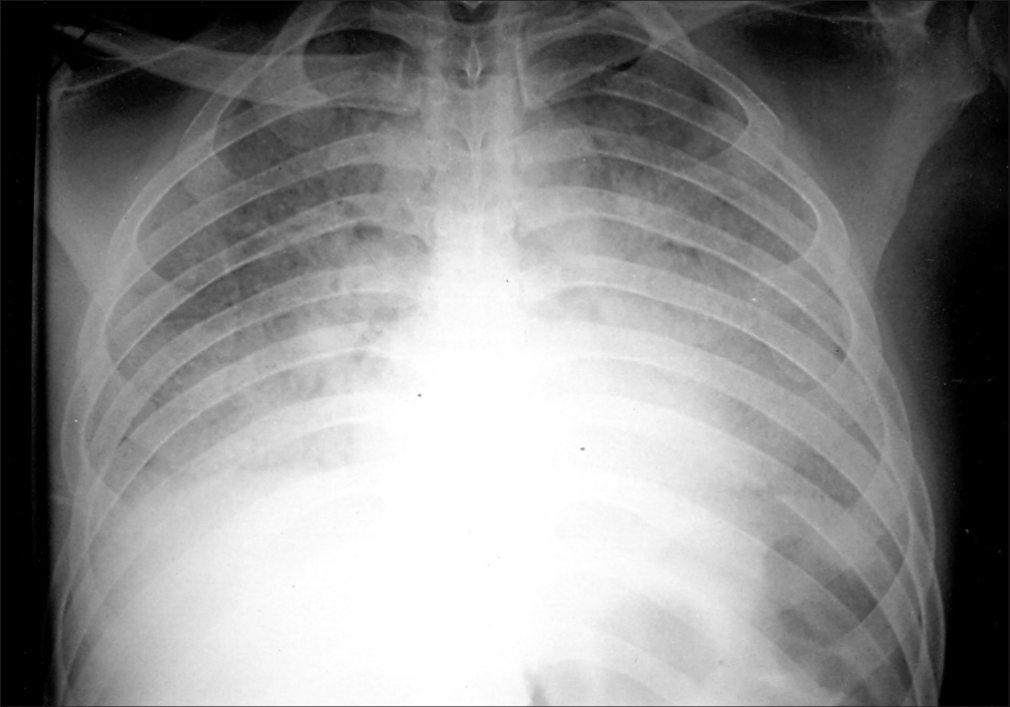
Chest radiograph showing bilateral diffuse reticulonodular infiltrations predominantly in perihilar and mid and lower zones

**Figure 2:**
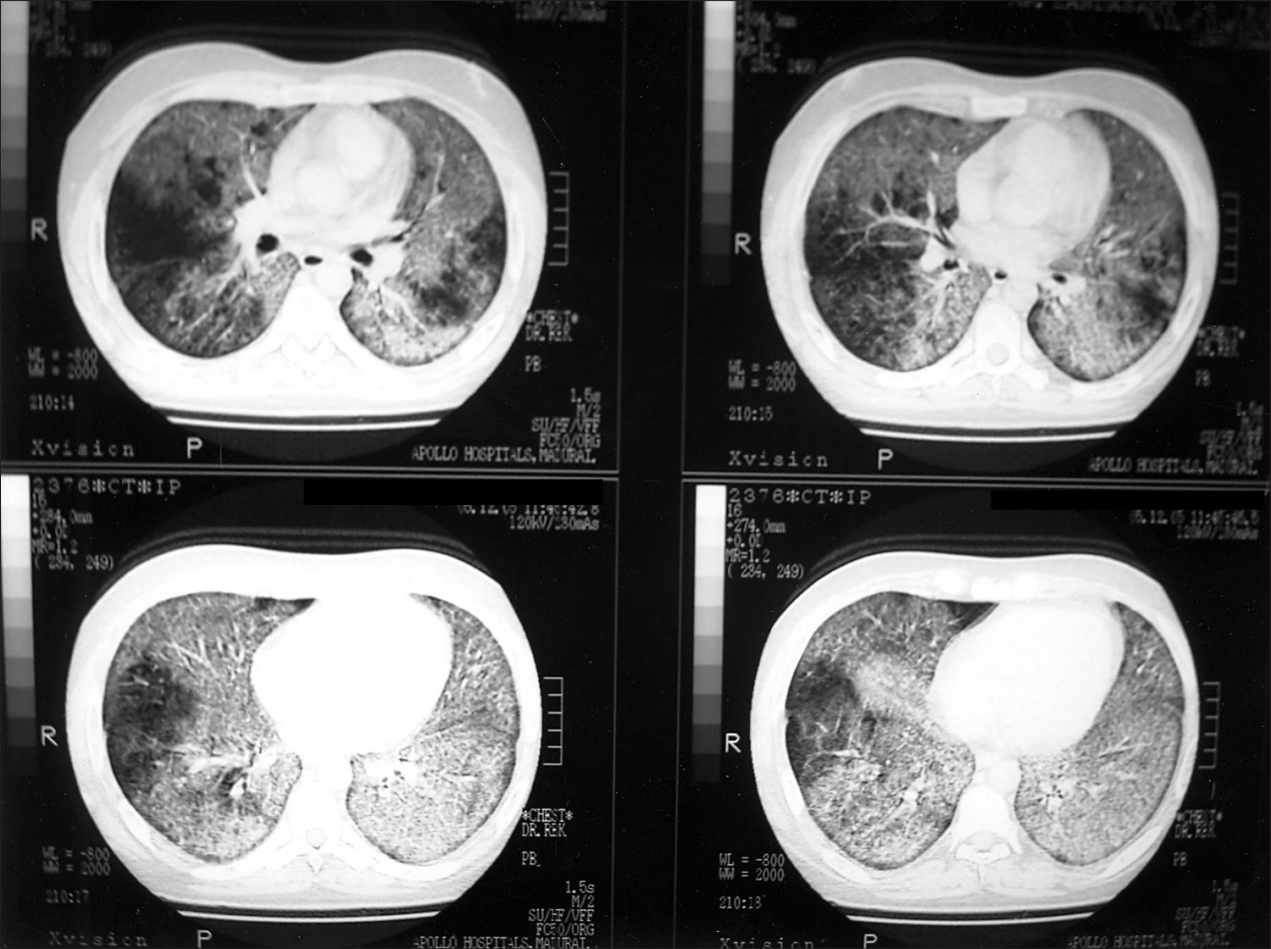
CT chest showing bilateral ground glass, “Crazy Paving” pattern

**Figure 3:**
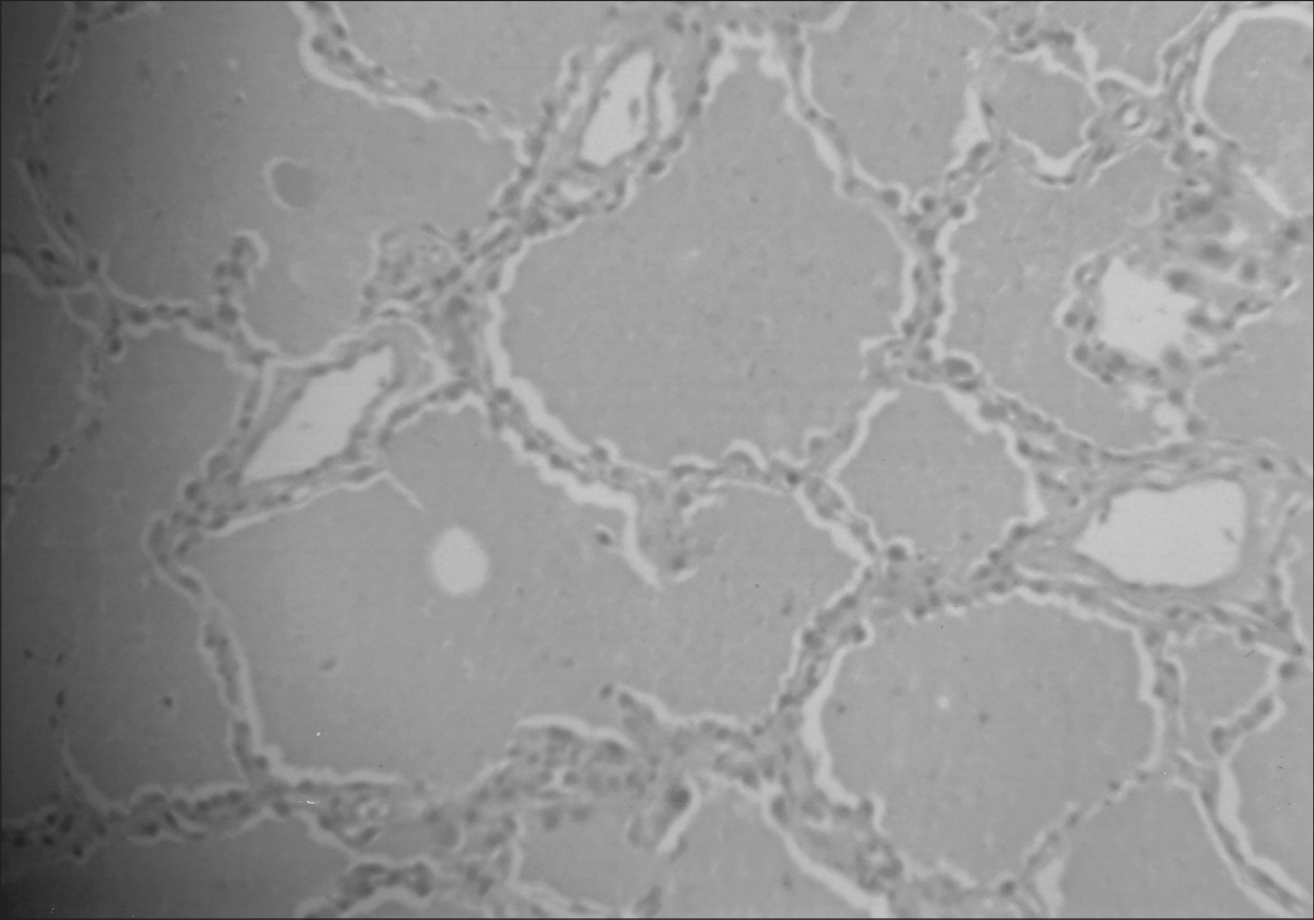
Photomicrograph of open lung biopsy showing eosinophilic material with granular appearance with positive periodic acid- Schiff stain

Blood tests showed Leucocytosis - 19,500 cells/cmm, DC-P 94%, L 2%, E 1%, M 1%. Hb-11.5 gm. ESR-87 mm/ hr. Coagulation profile, Liver Function Test and Renal Biochemistry were within normal limits. HIV and HbsAg was negative. Chest radiograph [Figure 1] showed bilateral diffuse reticulonodular pattern. High resolution computed tomography (HRCT) chest [Figure 2] showed bilateral “crazy paving” pattern of infiltration suggestive of Alveolar Proteinosis. Broncho alveolar lavage showed eosinophilic material with granular appearance with periodic acid- Schiff (PAS) stain positive. It was negative for malignant cells, AFB and fungal elements. Open lung biopsy [Figure 3] revealed eosinophilic secretions with granular appearance suggestive of alveolar proteinosis. Patient was subjected for bilateral sequential whole lung lavage.

**Figure 4:**
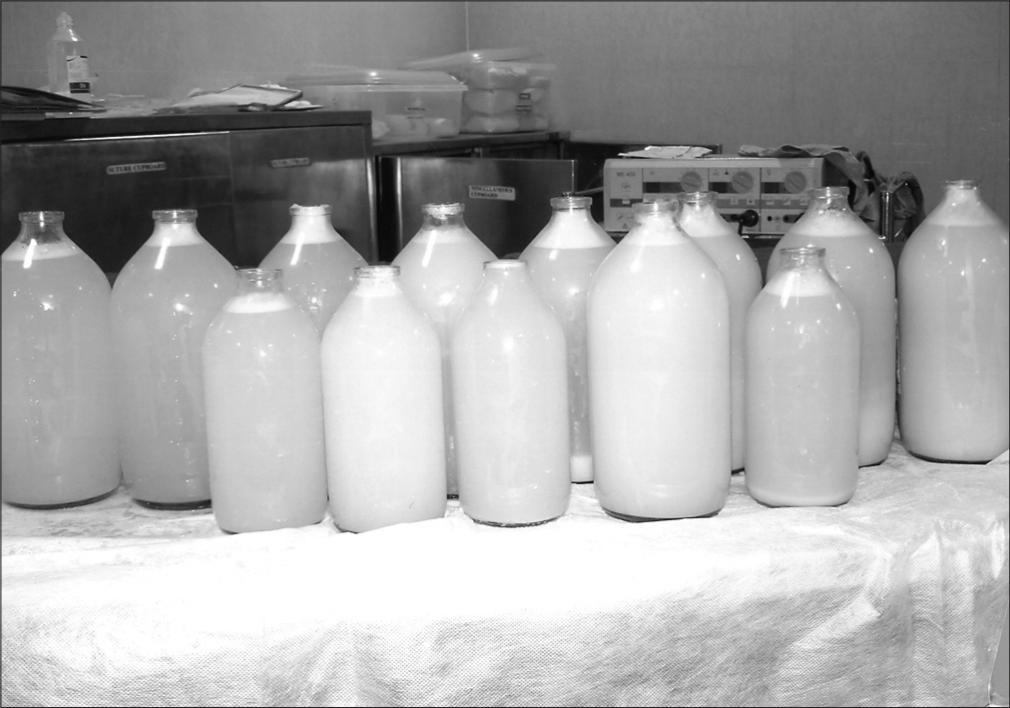
Whole lung lavage specimen containing milky and opalacent fluid

Whole lung lavage of the right lung was done under general anesthesia using double lumen endobronchial tube. A total of about 30 liters of warm saline was poured into the right lung and then suctioning of fluid done. Chest physiotherapy was done, to aid in dislodgement of secretions. Initial lavage fluid [Figure 4] was opalescent and milky white. The final bottle of lavage fluid collected was thin in consistency and clear. Patient was ventilated overnight and prepared for lavage of the left lung. Left Lung Lavage was done after a five-day interval. Post-lavage period was uneventful There was remarkable radiological clearance of lung opacities along with corresponding improvement in patient's oxygenation levels. His saturation was 97% at room air.

**Figure 5:**
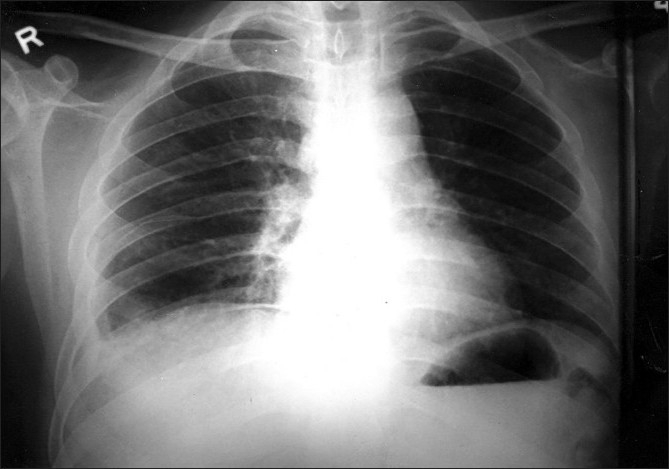
Post whole lung lavage chest radiograph, showing normal study

At the time of discharge, patient was comfortable at rest and off oxygen supplementation with no respiratory symptoms. Follow-up chest radiograph after two weeks showed clear lung fields [Figure 5].

## DISCUSSION

Pulmonary alveolar proteinosis (PAP) is a rare disorder of unknown etiology and variable natural course, which may range from respiratory failure and death to spontaneous resolution. It was first described by Rosen[1] in 1958 and till date less than 500 cases have been reported in literature. Pulmonary alveolar proteinosis is characterized by intra alveolar accumulation of lipid and proteinaceous material that is PAS positive when visualized on light microscopy. There is no evidence of inflammation and, typically, there is preservation of the lung architecture. It is clinically associated with increased work of breathing and derangement of gas exchange. There are three distinct types of PAP described as congenital, acquired and secondary-each with variable etiology, course, treatment and outcome. In congenital PAP, mutations occur in the genes encoding for surfactant protein B or C or the beta chain of the receptor for granulocyte colony stimulating factor.[2] Secondary Pulmonary alveolar proteinosis occurs in conditions with reduced number of alveolar macrophages or those with a functional impairment of macrophages. These include use of immunosuppressive agents, hematological cancers, exposure to toxic fumes or inorganic dusts like silica and infections which include *Nocardia* species, *Mycobacterium tuberculosis* and *Mycobacterium avium-intercellulare*, *Pneumocystis jerovici*, *Cytomegalovirus* and *Cryptococcus*. Acquired pulmonary alveolar proteinosis accounts for 90% of the cases; the median age of diagnosis is 39 years with a male -to - female ratio of 2.65:1.0. There is a history of smoking in 72% of patients.[3]

The most common clinical presentation of acquired pulmonary alveolar proteinosis is that of insidious onset, progressive dyspnea followed by cough, which may be dry or with white thick ‘gummy’ sputum.[4] Chest pain and hemoptysis are unusual symptoms. Weight loss and malaise are common but fever is uncommon. Presence of fever indicates secondary infection. Clinical examination findings can be unremarkable but there can be inspiratory crackles, cyanosis and clubbing, in late stages.

The chest radiograph usually reveals bilateral airspace disease with an ill defined nodular or confluent pattern. There may be a peri hilar predominance of ‘bat wing ’appearance of pulmonary edema but without other radiographic features of left sided heart failure.[5] It is to be noted that the extent of radiographic abnormalities is often disproportionately increased relative to the severity of the symptoms or the physical signs. HRCT of the chest shows the pattern of patchy, ground glass opacifications with superimposed interlobular thickening which is referred to as crazy paving.[6] This radiological pattern was found in our patient. The most common abnormal laboratory data is modest elevation of serum LDH.[7] The presence of fever and elevated total counts in our patient indicated presence of secondary infection.

The bronchoalveolar lavage specimen in a patient with pulmonary alveolar proteinosis is opaque with a milky appearance. There are large foamy macrophages or monocyte-like alveolar macrophages and increased number of lymphocytes and few other inflammatory cell types. There are large acellular eosinophilic bodies in diffuse background of granular material which stains with periodic acid - Schiff. There may be elevated levels of surfactant proteins A and D, but more studies are required to ascertain their specificity.[5,8] A definitive diagnosis of pulmonary alveolar proteinosis is most often based on tissue examination obtained by either transbronchial lung biopsy or open lung biopsy. The option of open lung biopsy had to be considered as transbronchial lung biopsy could not be done.

The clinical course of pulmonary alveolar proteinosis runs either a spontaneous remission as noted by Kariman *et al*. [9,10] and Larsen *et al.*[9,10] or a progressive deterioration in condition resulting in high mortality rates.[11,12]

Whole lung lavage or sequential lung lavage is an accepted method of treatment that has been historically described by Ramirez-Rivera.[13] It is a moot point whether this Whole lung lavage would completely relieve the patient of this disease, as it has been noted in one of the studies[4] that they needed repetition of the procedure. There have been anecdotal reports of response following GM-CSF therapy[14,15] and lung transplantation[16] although they cannot be suggested as a modality of treatment.

The purpose of this case report is to highlight the need for a high index of suspicion to diagnose this eminently diagnosable and treatable illness. There should be no hesitation in resorting to invasive procedures like open lung biopsy and prolonged lavage under general anesthesia.
